# The role of environmental vs. biotic filtering in the structure of European ant communities: A matter of trait type and spatial scale

**DOI:** 10.1371/journal.pone.0228625

**Published:** 2020-02-19

**Authors:** Olga Boet, Xavier Arnan, Javier Retana

**Affiliations:** 1 CREAF, Bellaterra (Cerdanyola del Vallès), Catalonia, Spain; 2 Universitat Autònoma de Barcelona, Cerdanyola del Vallès, Catalonia, Spain; University of Waikato, NEW ZEALAND

## Abstract

Functional trait-based approaches are increasingly used for studying the processes underlying community assembly. The relative influence of different assembly rules might depend on the spatial scale of analysis, the environmental context and the type of functional traits considered. By using a functional trait-based approach, we aim to disentangle the relative role of environmental filtering and interspecific competition on the structure of European ant communities according to the spatial scale and the type of trait considered. We used a large database on ant species composition that encompasses 361 ant communities distributed across the five biogeographic regions of Europe; these communities were composed of 155 ant species, which were characterized by 6 functional traits. We then analysed the relationship between functional divergence and co-occurrence between species pairs across different spatial scales (European, biogeographic region and local) and considering different types of traits (ecological tolerance and niche traits). Three different patterns emerged: negative, positive and non-significant regression coefficients suggest that environmental filtering, competition and neutrality are at work, respectively. We found that environmental filtering is important for structuring European ant communities at large spatial scales, particularly at the scale of Europe and most biogeographic regions. Competition could play a certain role at intermediate spatial scales where temperatures are more favourable for ant productivity (i.e. the Mediterranean region), while neutrality might be especially relevant in spatially discontinuous regions (i.e. the Alpine region). We found that no ecological mechanism (environmental filtering or competition) prevails at the local scale. The type of trait is especially important when looking for different assembly rules, and multi-trait grouping works well for traits associated with environmental responses (tolerance traits), but not for traits related to resource exploitation (niche traits). The spatial scale of analysis, the environmental context and the chosen traits merit special attention in trait-based analyses of community assembly mechanisms.

## Introduction

A central goal in ecology is to understand the processes underlying community assembly [1–4]. A plethora of processes (e.g. evolutionary history, environmental constraints, biotic interactions) operating at different spatial and temporal scales contribute to community assembly patterns [5]. Environmental filtering is the first filter that selects a subset of species from the regional species pool [6,7]. It is primarily determined by evolutionary and historical factors, as well as dispersal barriers [8]. In turn, species interactions (i.e. biotic filtering) can exert a strong influence on the final number of species that co-occur in a community, mainly through competitive exclusion [9]. On those taxa where interspecific competition might be an important process structuring local assemblages, it is particularly useful to understand the relative contribution of environmental filtering vs. competition [2,10]. A relevant point is not which mechanism is in operation, but which mechanism has the strongest influence on communities under particular conditions. The relative influence of different assembly rules might depend on the spatial and temporal scales of analysis [10] and the environmental context [11,12]. While environmental filtering is assumed to be strongest at the regional scale [13–15], species interactions (i.e. competition) predominate at local spatial scales [16,17]. Meanwhile, environmental filtering is expected to dominate during early succession [18] and high elevations [12,19,20] where environmental conditions are harshest, with biotic filtering becoming increasingly important in the later stages of succession [21].

Functional traits provide important insights into mechanisms of community assembly [13,21–24]. A given environmental condition selects for individuals with similar traits, meaning particular traits are necessary or better adapted to that condition. Thus, environmental filtering is predicted to result in local communities comprised of species with similar functional traits that allow species to persist (‘functional clustering’) [3]. On the other hand, if functionally similar groups of individuals compete more intensively with one another than functionally dissimilar species (‘competitive exclusion principle’, [9]), this allows functional dissimilar individuals to coexist (‘limiting similarity principle’, [25]). Consequently, biotic filtering is predicted to result in a local community with different functional traits (‘functional over-dispersion’). Moreover, neutral theory assumes that species coexist and persist in a system independently of their traits [4]. These three mechanisms (environmental filtering, biotic filtering and neutral theory) might even co-occur simultaneously and blur the patterns, or they might occur sequentially along environmental gradients [23].

An important issue to consider when testing for assembly processes using functional traits is that, depending on the type of functional traits we use, different patterns might emerge [11,22,26]. Some functional traits clearly relate to environmental responses (‘ecological tolerance traits’) [27], and they might only respond to environmental filtering rather than to competition processes. In these cases, assembled species might show functional clustering. Other traits relate to the way species exploit resources (‘ecological niche traits’) and so they lead to species exclusion or niche segregation as a result of competition processes [11]. These traits might not respond to environmental filtering, so species could show functional over-dispersion. Finally, traits directly related to the species’ competitive abilities to exploit one or a few limited resources (‘competitive abilities traits’) might be selected by competition processes (and not by any environmental filter). This is because they provide better fitness and then, similarly to when environmental filtering is at work, functional clustering instead of functional over-dispersion might be found [11,26]. When multiple trait dimensions are considered, the ecological differentiation between species may be elucidated, because these sets of traits are likely to be relevant to the ecological tolerance, ecological niche and competitive ability of a species [28]. This can be even more complex when there are positive or negative correlations among traits linked to tolerance and competitive outcomes [11].

Ants are a suitable model system when it comes to analysing community assembly processes. Ants are among the most diverse, abundant and ecologically relevant organisms on earth [29]. They are highly sensitive to environmental change along climatic [30], habitat [31], productivity [32] or disturbance gradients [33]. Ants, like plants and other sessile organisms, have the ability to monopolize space and other resources, and therefore influence other species in the areas they occupy [34]. Thus, competitive interactions have been widely reported among ant species, which have usually been organized into behavioural dominance hierarchies [33,35]. Even though competition is one of the most addressed topics in myrmecological research, there is still a contentious debate about the role of competition in structuring ant communities [36–39]. Meanwhile, research in ant functional ecology has made important progress in recent years. Nowadays, we have different ant trait databases covering a wide range of traits (e.g. morphological, behavioural, physiological and life-history traits) for thousands of ant species [27,40–43]. Interestingly, many studies have demonstrated that several functional traits act as the link between ant species distribution and the environment [27,33,42,44,45], i.e. they are ‘ecological tolerance traits’ that might be influenced by environmental filtering processes. Meanwhile, other functional traits relate to resource exploitation [46], i.e. they are ‘ecological niche traits’ or ‘competitive ability traits’ that might be more sensitive to competition rather than to environmental filtering.

Through the analysis of functional divergence and co-occurrence patterns between pairs of ant species, we investigate the contexts that make some assembly rules more influential than others in structuring ant communities. Specifically, we investigate how these rules vary according to the scale of analysis (local, regional and global) and the type of functional trait considered. To address this topic, our trait-based approach takes advantage of a large database on ant species composition that encompasses 361 ant communities distributed across the five most representative biomes of Europe, which clearly differ in climatic, physical and historical conditions [41]. These communities were composed of a total of 155 species, characterized by functional traits that were grouped according to their functional role. Aware of the importance of the choice of functional traits when seeking to meet the objectives of the study [7,11,21], we have only characterized species according to ecological tolerance and ecological niche traits, and not to competitive ability traits. Thus, similar patterns are obtained (functional clustering) despite the fact that they have been generated by different processes (i.e. environmental or biotic filtering) when using ecological tolerance and competitive ability traits [7]. Our specific objectives are: 1) to analyse the relative role of environmental filtering and competition on the structure of European ant communities at local, biogeographic and continental scales; and 2) to analyse whether the type of functional trait plays a relevant role in determining the relative importance of environmental filtering and competition. We hypothesize that the relative role of environmental filtering and competition depends on: a) the spatial scale, that is to say, environmental filtering surpasses competition at larger spatial scales (i.e. Europe and biogeographic regions), while competition exceeds environmental filtering at local spatial scales; and b) ant functional trait type, that is to say, the analyses with ‘ecological tolerance traits’ will relate to environmental filtering processes, while the analyses with ‘ecological niche traits’ will be more related to competition processes.

## Materials and methods

### Ant community data

**Fig 1:**
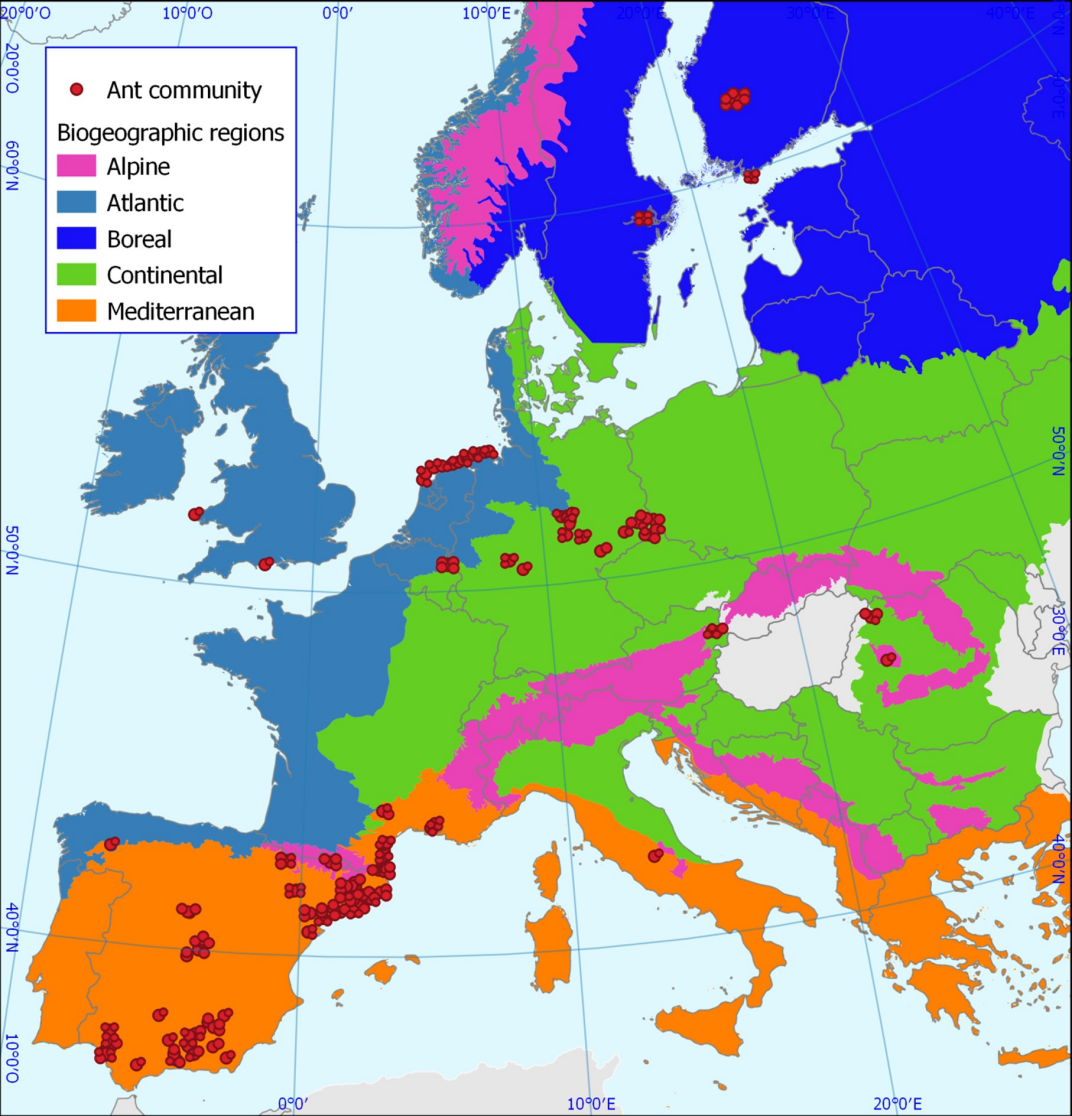
Map of the study area showing ant community distribution across the five biogeographic regions of Europe. EEA is original source of the biogeographical regions map Copyright holder: Council of Europe (CoE), Directorate-General for Environment (DG ENV). (https://www.eea.europa.eu/legal/copyright).

To measure co-occurrence patterns between pairs of ant species, first we assembled species composition data of local ant communities in Western Europe, which allowed us to perform analyses at different spatial scales: continental (hereafter, European scale), biogeographic and local scales. We considered the five most representative biogeographic regions of Europe (Mediterranean, Continental, Atlantic, Boreal and Alpine), which clearly differ in their climatic and historical conditions [41]. These ant communities comprised a total of 155 ant species, belonging to 29 genera and 5 subfamilies. Beforehand, we removed two ant species (*Solenopsis* sp., *Lasius neglectus*) from the original database (containing 157 ant species), since we did not obtain functional data for most traits analysed. At the European and biogeographic scales, we used the 361 local ant communities which were distributed across five biogeographic regions: Mediterranean (211 communities, 127 species), Continental (71, 51), Atlantic (27, 44), Boreal (29, 31) and Alpine (23, 27) (Fig 1). This database includes primary data collected during the author’s own field work in the past and data derived from an exhaustive search of the scientific literature (see 27,41,46 for more details on this database). We focused our analyses on presence-absence data. Therefore, this database was composed of six species x site matrices with occurrence data (one for Europe, and one for each of the five biogeographic regions). At the local scale (i.e., the scale of each local community), we looked for communities in which the occurrences of species were not at the whole plot level, but where there was data at the level of different baits within the plot. Overall, we used 24 local ant communities (also included in the general database) from the Mediterranean region, which encompassed 37 ant species. These communities were compiled from our own field work [31,45]. In each site, four-five series of five-six baits each were laid randomly over the entire study area (with 5-m spacing between two adjacent baits and also between series) for a total of four-six sampling days of 24 hr each. Baits were plastic discs, each of them with a different large food reward attractive to ants that tried to cover a wide range of potential types of food for ants. Each hour of every 24-hr sampling period, the identity of each ant species feeding at each bait was noted. This database contained 24 species x trap matrices with occurrence data at different baits (between 10 and 60) within a plot. We tried to obtain other databases from the other biogeographic regions in order to perform local scale analysis. However, we managed no more than a few localities in each region, and so we were unable to extend the analysis locally to regions other than the Mediterranean.

### Trait data

The 155 ant species were characterized according to six functional traits that clearly relate to species responses to environmental change (‘ecological tolerance traits’) or to competition (‘ecological niche traits’) (Table 1). Information about traits was obtained from the literature (see 27,33,41,46). It is worth noting that since ants are social, functional traits have been quantified at both the level of individual worker and that of the colony. The following traits were used: (i) ecological tolerance traits: number of queens, colony size (log-transformed) and brood cycle; and (ii) ecological niche traits: diet, diurnality and worker size. Trait characterization and functional significance is provided in Table 1. In order to check for highly correlated traits, we first carried out pairwise Pearson correlations among all traits. Most correlations were significant (p < 0.05), but with Pearson r coefficients < 0.7 (S1 Table). We then assumed that our chosen traits were not highly correlated and all of them were included in further analyses.

**Table 1:** Ant functional traits used in this study assembled in two main groups as regards they are ‘ecological tolerance’ or ‘ecological niche’ traits. A trait description, data type, empirical evidences to categorize traits as response or ecological niche traits are shown.

Group of traits / Functional trait	Description	Data type	Empirical evidences
**(A) Ecological tolerance**			Ants have achieved great ecological success due to their social structure. As other social organisms, the response of ant species to the environment is mainly conditioned by the morphological specialization and flexibility of castes within colonies.
• Number of queens	Monogynous (one queen), polygynous (more than one queen), or both	Qualitative	Polygyny tends to be correlated with abundant resources that permit rapid growth, enabling species to benefit from productive resource environments in ways unavailable to monogynous species.
• Colony size	The mean number of workers per colony	Quantitative	Colony size has a clear impact on resource exploitation because large colonies are competitively superior to small colonies by sending out more workers to collect food resources or battle neighbors.
• Brood cycle	Species with larvae within the nests from March to September, or species with larvae within the nests during the whole year	Qualitative	The annual cycles of ants, characterized by the presence or not of hibernating brood, are well adapted to the climate environments where they live.
**(B) Ecological niche**			Resource exploitation in ants aligns along two main trait dimensions of competitive interactions: one related to behavioral dominance, associated with large colony size, presence of multiple nests per colony and a collective foraging strategy, and another related to resource partitioning along dietary and microhabitat lines.
• Diet	Proportion of seeds, insect corpses, and liquid foods in diet	Qualitative (Fuzzy-coded)	The type of resources and the size of these resources are considered important in mediating competition for resources among ant species.
• Diurnality	Strictly diurnal or non-strictly diurnal species	Qualitative	In ant communities, subordinate species are subject to interspecific competition, although they can avoid dominants by being less temperature-limited and foraging at different temperatures.
• Worker size	Distance from the tip of mandibles to the tip of the gaster (mm)	Quantitative	Worker size may constrain where and when ants are able to forage, as it is a prominent characteristic that affects all aspects of insect physiology.

See S1 File for the bibliographic sources.

### Data analyses

Different trait-based approaches have been used to distinguish the stochastic and deterministic (environmental vs. biotic filtering) processes that structure biotic communities. The approach we use can disentangle the role of environmental filtering and competitive exclusion by analysing the relationship between species pair co-occurrence and functional dissimilarity [2]. From this analysis, three different patterns might emerge. First, if species with similar functional traits co-occur more often than expected by chance, the relationship between co-occurrence and functional dissimilarity of pairs of species will be significant and negative (i.e. environmental filtering process). Contrary to this, if species with divergent traits co-occur more often than expected at random, the relationship will be significant and positive (i.e. competitive exclusion process). Finally, non-significant relationships between co-occurrence and functional dissimilarity of species pairs are also possible (i.e. neutral theory processes). This would be the case where species co-occur independently of their functional similarity, or alternatively, if environmental filtering and competition exclusion are simultaneously at work with similar contributions. Here, we assume that two species co-occur when they occur spatially in the same community, although they might not share the same foraging time.

The co-occurrence index for each species pair was calculated within each species x site (European and regional scales) and species x bait (local scale) matrix. Data for the co-occurrence analyses consist in binary presence-absence matrices, where each row was a species, each column a site (or a bait), and the entries were presence (1) or absence (0) of a species in a site or a bait. Pairwise co-occurrence was calculated using the Jaccard index of similarity (JIab) for each pair of species in each matrix [47]:

JIab=AB÷(A+B+AB)

where A and B are the number of sites where only species *a* and species *b* occur, respectively, and AB the number of sites where species *a* and *b* co-occur. The Jaccard similarity index takes values between 0 and 1, where 0 means that the two species are never found in the same site, and in our case, that co-occurrence is null; while 1 indicates that the two species are always together, and in our case, that the co-occurrence is total.

In order to measure functional dissimilarity between species pairs, we computed Gower’s dissimilarity between two species based on each functional trait separately, pooling traits according to whether they are ‘ecological tolerance’ or ‘ecological niche’ traits, and pooling all traits together. We used Gower’s dissimilarity, so that we would be able to deal with quantitative and qualitative traits [48]. To compute it, we used a functional matrix where rows were species, columns were traits, and cell values were the trait values. Since Gower's dissimilarity depends on the number of species in the matrix, it was only calculated for each pair of species with data from the largest scale (Europe) where the number of species is highest. For each pair of species, nine functional dissimilarities were calculated: one with all functional traits together; one with only the ecological niche traits; one with the ecological tolerance traits; and one for each of the six traits separately. For these computations we used the ‘vegan’ [49] and ‘cluster’ [50] packages in R software v. 3.2.2 [51].

The relationship between the functional dissimilarity and the co-occurrence index between species pairs was tested by using linear models. Given the large number of zeros in the co-occurrence index and failure to meet the normal assumptions, we carried out the analyses in two steps. First, we transformed the co-occurrence index into a binary variable indicating whether or not there was occurrence of the pair of species in each matrix. We used a generalized linear model with a binomial distribution and a logit link function to perform the analysis (hereafter, binary co-occurrence analysis). In a second step, we applied a general linear model to make the model with the co-occurrence index where the pair of species occur at least once in the matrix (hereafter, co-occurrence strength analysis). In this case, the co-occurrence index was log-transformed to satisfy normality assumptions. We performed 18 analyses at the European scale (nine analyses for binary occurrence matrices and nine for co-occurrence strength matrices, these last nine comprising one analysis with all traits together, two analyses corresponding to each group of traits, and six analyses corresponding to each trait separately), 90 analyses at the biogeographic scale (forty-five for binary occurrence matrices and forty-five for occurrence strength matrices, of which nine analyses corresponded to each of the five biogeographic regions), and 333 analyses at the local scale (117 for binary occurrence matrices and 216 for co-occurrence strength matrices, comprising 37 analyses with all traits together, 37 for each group of traits and 222 for each singular trait). It is worth noting that binary co-occurrence analyses were only performed in locations where more than five pairs of species showed values of co-occurrence = 0. Generalized and general linear models were conducted using the ‘stats’ package in R.

## Results

**Fig 2:**
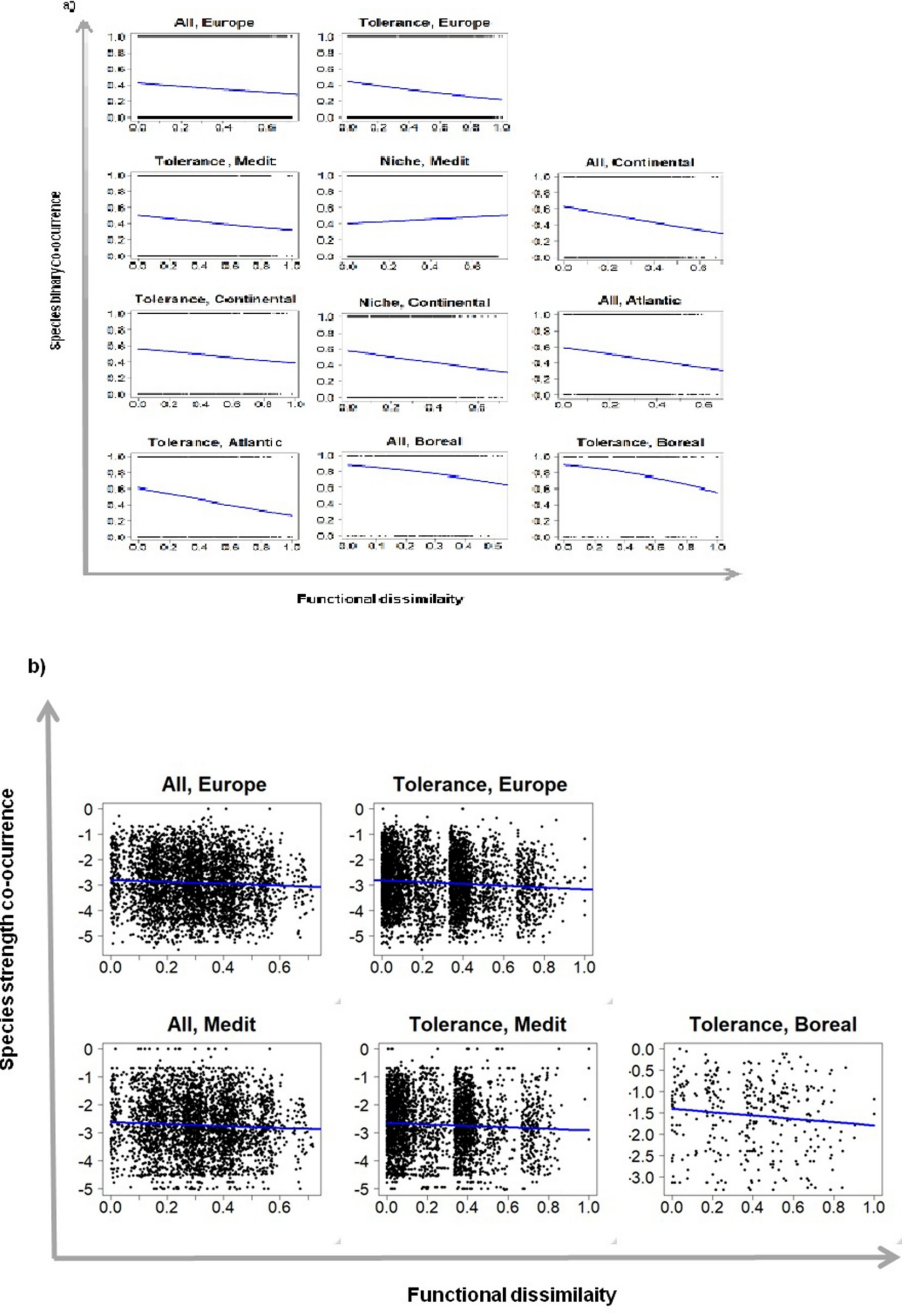
Relationships between species co-occurrence and functional dissimilarity. We show the significant relationships between binary co-occurrence (a) or co-occurrence strength (b) and functional dissimilarity for all traits together, ecological tolerance traits, ecological niche traits between pairs of species at the European and biogeographic region scales. Abbreviations: All, all traits together; Tolerance, ecological tolerance traits; Niche, ecological niche traits; Medit, Mediterranean.

Our analyses revealed different patterns in the relationship between co-occurrence and functional dissimilarity among pairs of species according to the scale of analysis and the type of trait used (Table 2). At the European scale, results are consistent between binary co-occurrence and co-occurrence strength analyses. Both analyses showed significant and negative model coefficients when functional dissimilarity was computed with all traits together and with ecological tolerance traits, but non-significant model coefficients when functional dissimilarity was computed with ecological niche traits (Table 2 and Fig 2A and 2B).

**Table 2:** Statistical outputs from the binary co-occurrence and co-occurrence strength analyses analyzing the relationship between species co-occurrence and functional dissimilarity between species pairs at the Europe and biogeographic region scales. Different analyses were conducted for functional dissimilarity computed with all traits together, ecological niche traits and ecological tolerance traits.

	Binary co-occurrence analysis				Co-occurrence strength analysis			
	Value	Std. Error	z value	Pr(>|z|)	Value	Std. Error	t value	Pr(>|t|)
**All traits together**								
Europe	**-0.8536**	**0.1298**	**-6.5750**	**<0.0001**	**-0.3521**	**0.1082**	**-3.252**	**0.0011**
Mediterranean	-0.1713	0.1510	-1.1340	0.2566	**-0.3545**	**0.1162**	**-3.051**	**0.0023**
Continental	**-2.0021**	**0.3938**	**-5.0840**	**<0.0001**	-0.0632	0.2064	-0.306	0.7590
Atlantic	**-1.7249**	**0.5082**	**-3.3940**	**0.0007**	0.2528	0.4132	0.612	0.5410
Boreal	**-2.8099**	**0.9675**	**-2.9040**	**0.0037**	-0.3840	0.3475	-1.105	0.2700
Alpine	-0.7934	0.8098	-0.9800	0.3270	-0.0595	0.3916	-0.152	0.8790
**Ecological tolerance**								
Europe	**-1.0475**	**0.0818**	**-12.8010**	**< 0.0001**	**-0.4323**	**0.0703**	**-6.146**	**<0.0001**
Mediterranean	**-0.7722**	**0.1001**	**-7.7180**	**<0.0001**	**-0.2542**	**0.0790**	**-3.215**	**0.0013**
Continental	**-0.7253**	**0.2246**	**-3.2290**	**0.0012**	-0.0948	0.1200	-0.791	0.4290
Atlantic	**-1.4734**	**0.2869**	**-5.1360**	**<0.0001**	-0.1783	0.2360	-0.756	0.4500
Boreal	**-2.0166**	0.4985	-4.0450	0.0001	**-0.3851**	**0.1805**	**-2.134**	**0.0335**
Alpine	-0.5906	0.5046	-1.1700	0.2420	-0.0781	0.2384	-0.328	0.7430
**Ecological niche**								
Europe	0.1500	0.1034	1.4500	0.1470	0.0344	0.0851	0.404	0.6860
Mediterranean	**0.5349**	**0.1224**	**4.3700**	**0.0000**	-0.1434	0.0932	-1.538	0.1240
Continental	**-1.4792**	**0.3542**	**-4.1760**	**0.0000**	0.0996	0.2031	0.49	0.6240
Atlantic	-0.0368	0.4746	-0.0770	0.9380	0.5858	0.3736	1.568	0.1180
Boreal	-0.6189	1.0260	-0.6030	0.5460	0.2701	0.3820	0.707	0.4800
Alpine	-0.2786	0.7469	-0.3730	0.7090	0.0020	0.3799	0.005	0.9960

Significant coefficients (p<0.05) are shown in bold.

At the regional scale, binary co-occurrence and co-occurrence strength analyses revealed consistent and complementary results. When analyses were based on all traits together, binary co-occurrence analyses showed significant and negative estimates for the Boreal, Continental and Atlantic regions (from the steepest to the lowest slopes) (Fig 2A); complementarily, co-occurrence strength analysis showed a significant and negative estimate for the Mediterranean region (Table 2 and Fig 2B). When using ecological tolerance traits, both binary co-occurrence and co-occurrence strength analyses revealed significant and negative model estimates for the Mediterranean and Boreal regions (Fig 2A and 2B); additionally, the binary co-occurrence analyses showed significant and negative estimates for the Continental and Atlantic regions (Table 2 and Fig 2A). With regard to analyses based on ecological niche traits using the binary co-occurrence approach, significant model estimates were only found for the Mediterranean (positive estimate) and Continental (negative estimate) regions (Table 2). In short, for those biogeographic regions where the relationship between co-occurrence and functional dissimilarity was significant regardless of the analysis employed, all relationships were negative when using all traits together or ecological tolerance traits to compute functional dissimilarity, while relationships did not have a consistent pattern when ecological niche traits were used.

At the local scale, of the one hundred and eleven model coefficients, only eleven were significant (Table 3). Specifically, binary co-occurrence analyses only revealed a significant and positive model coefficient in one site, and this when computing functional dissimilarity with ecological tolerance traits. Meanwhile, co-occurrence strength analyses revealed several significant model coefficients. When functional dissimilarity was computed with all traits together, only four sites depicted significant relationships (three negative and one positive); when computed with ecological tolerance traits, only two sites depicted significant relationships (one positive and one negative); and when computed with ecological niche traits, only four sites depicted significant relationships (all negative).

**Table 3:** Estimated coefficients from the analyses between functional dissimilarity computed with all traits together, ecological niche traits and ecological tolerance traits, and binary co-occurrence as well as co-occurrence strength between pairs of species at the local scale.

			Binary co-occurrence analysis			Co-occurrence strength analysis		
Locality	n	n0	All together	Ecological tolerance	Ecological niche	All together	Ecological tolerance	Ecological niche
loc.01	45	11	1.5660	0.9093	0.8339	1.6440	**1.7331**	0.4290
loc.02	120	40	1.3924	1.6622	-0.0853	**1.2182**	0.5188	0.7802
loc.03	10	0	-	-	-	**-6.7295**	-3.6431	-3.7509
loc.04	66	29	-0.8883	1.5651	-1.9334	**-1.9270**	**-1.2384**	**-1.4091**
loc.05	6	0	-	-	-	**-9.2907**	-1.5765	-2.5890
loc.06	21	12	8.3800	-0.7843	7.9885	-5.3871	-1.3070	-2.1909
loc.07	21	0	-	-	-	-0.9864	-1.6160	0.2878
loc.08	21	6	-2.6100	-3.7100	-0.5150	0.1842	-0.5069	0.2548
loc.09	120	40	2.9945	0.6239	2.2770	-0.1972	0.3136	-0.4402
loc.10	28	7	5.9686	-3.4477	5.6574	0.1872	0.7015	-0.1607
loc.11	66	20	-0.7810	**6.2998**	-2.7330	0.4620	-0.0859	0.5016
loc.12	55	3	-	-	-	0.4804	0.6585	0.0097
loc.13	21	3	-	-	-	-1.8155	1.2629	**-2.3216**
loc.14	28	4	-	-	**-**	-0.7276	1.1398	**-1.1046**
loc.15	21	6	4.3511	4.2968	1.3112	-0.8123	0.8628	**-1.8472**
loc.16	21	0	-	-	-	-0.3357	0.0022	-0.4171
loc.17	28	1	-	-	-	1.4501	1.2610	0.6379
loc.18	36	2	-	-	-	0.4246	1.2738	-0.4789
loc.19	15	6	-3.7120	-5.9210	-1.3378	-0.8853	-0.1946	-0.9405
loc.20	55	35	1.0030	1.6240	-0.5537	-0.0839	0.5128	-0.7753
loc.21	36	20	1.1635	2.2606	-0.8901	-1.1148	-0.5762	-0.9014
loc.22	28	4	-	-	-	-0.0444	-0.2654	0.1023
loc.23	66	3	-	-	-	0.2591	0.6642	-0.1928
loc.24	45	13	2.1939	-0.7670	2.8689	0.5320	-0.0018	0.5528

Significant coefficients (p<0.05) are shown in bold. Abbreviations: n, number of species pairs in each locality (loc.); n0, number of species pairs with co-occurrence = 0 in each locality. Note that binary co-occurrence analyses were only performed when at least six pairs of species depicted values of co-occurrence = 0.

When analyses were performed for each single trait at the European and biogeographic region scales, we also found different patterns according to the scale of analysis and type of trait. (Table 4). At the largest scale (Europe), binary co-occurrence analyses revealed significant and negative coefficients for the three ecological tolerance traits (i.e. number of queens, colony size and brood cycle), but no significant coefficients for any of the ecological niche traits (i.e. diet, diurnality and worker size). Complementarily, co-occurrence strength analyses showed significant and negative estimates for number of queens and colony size (ecological tolerance traits), and significant and positive model coefficients for diurnality and worker size (ecological niche traits) (Table 4). At the intermediate spatial scale (biogeographic region scale) and regardless of the analytical approach, significant estimate models were always negative for the three ecological tolerance traits, but negative or positive for ecological niche traits depending on the trait and biogeographic region (Table 4). Thus, when functional dissimilarity was based on the number of queens, binary co-occurrence analyses revealed significant estimates for the Mediterranean, Continental and Atlantic regions; meanwhile, co-occurrence strength analysis revealed significant estimates for the Mediterranean, Atlantic and Boreal regions. With respect to colony size, significant relationships were found for the Boreal region from both binary co-occurrence and co-occurrence strength analyses, and for the Mediterranean region from the binary co-occurrence analysis alone. When using brood cycle, binary co-occurrence analyses revealed significant estimates for all regions except the Alpine, while co-occurrence strength analyses showed no significant estimate. Moving to the ecological niche traits, when functional dissimilarity was based on diet, binary co-occurrence analyses revealed positive estimates for the Mediterranean and Alpine regions, and negative estimates for the Continental region; meanwhile, co-occurrence strength analyses showed a significant negative effect for the Mediterranean region. When using diurnality, positive and negative estimates were found for the Mediterranean and Alpine regions, respectively, from binary co-occurrence analyses, while co-occurrence strength analyses did not find any significant relationship in any region. Finally, when analyses were based on worker size, binary co-occurrence analyses showed a significant positive estimate for the Mediterranean region, but significant negative estimates for the Continental and Atlantic regions; meanwhile, co-occurrence strength analyses only showed a positive estimate, particularly for the Atlantic region (Table 4).

**Table 4:** Estimated coefficients from the analyses between co-occurrence (binary co-occurrence and co-occurrence strength) and functional dissimilarity between species pair based on single traits at the European and biogeographic region scales.

	Europe	Mediterranean	Continental	Atlantic	Boreal	Alpine
**Binary co-occurrence**						
**Ecological tolerance traits**						
Number of queens	**-0.3644**	**-0.3019**	**-0.2148**	**-0.4001**	-0.4355	-0.2395
Colony size	**-1.1945**	**-0.9277**	-0.2475	-0.7237	**-2.4034**	-0.8136
Brood cycle	**-0.3034**	**-0.1986**	**-0.2649**	**-0.5512**	**-0.5571**	-0.1761
**Ecological niche traits**						
Diet	-0.0327	**0.1731**	**-1.1904**	0.2276	-1.2760	**1.4605**
Diurnality	0.1243	**0.2137**	-0.1336	-0.0061	-0.0687	**-0.5479**
Worker size	0.0959	**0.3546**	**-0.8617**	**-1.0333**	0.7673	0.1225
**Co-occurrence strength**						
**Ecological tolerance traits**						
Number of queens	**-0.2701**	**-0.1805**	-0.0041	**-0.2344**	**-0.3313**	-0.2478
Colony size	**-0.3730**	-0.2393	-0.0160	-0.1242	**-0.7977**	0.6684
Brood cycle	-0.0324	0.0034	-0.0587	0.0686	0.0228	0.0556
**Ecological niche traits**						
Diet	-0.0967	**-0.2379**	0.2596	0.1721	-0.4340	-0.0237
Diurnality	**0.0655**	0.0465	-0.0370	0.1065	0.1534	0.0066
Worker size	**0.1917**	0.1465	-0.2232	**0.9352**	0.1889	0.1269

Significant coefficients (p<0.05) are shown in bold.

When analyses were performed for each single trait at the local scale, we only found a few significant coefficients. Of the two hundred and twenty-two estimated coefficients, only eighteen were significant, these breaking down into five positive (all for tolerance traits) and thirteen negative (four for ecological tolerance traits and nine for ecological niche traits) (S2 Table).

## Discussion

Our first objective was to analyse the relative role of environmental filtering and competition on the structure of European ant communities at different spatial scales. We partially corroborated our first hypothesis, because our results suggest that environmental filtering is the main mechanism influencing ant community structure at the largest spatial scale (European). Meanwhile, different patterns emerge at the intermediate spatial scale depending on the biogeographic region (although environmental filtering is the main mechanism at play), but no ecological mechanism (environmental filtering or competition) is more important than the other at the local scale in the Mediterranean region. Overall, as the scale of analysis increases, environmental filtering becomes more important as a structuring mechanism for European ant communities.

When we analyse assembly rules at the continental scale, we encounter more widely contrasting environmental conditions than at smaller spatial scales, such as different habitat types and even biogeographic regions. Thus, the larger the scale, the more likely it is that more widely contrasting environmental conditions will be found. Species inhabiting environments with severe environmental conditions must adapt morphologically, behaviourally and/or ecologically to the harsh conditions. Therefore, species co-occurring in similar harsh habitats have more functional similarities in terms of their traits that will allow them to persist in those conditions when compared with species living in different environmental conditions [3,20].

When we go down to smaller spatial scales, high spatial heterogeneity with very widely contrasting conditions is less likely to be found. At the smallest spatial scale we analysed, local scale, and contrary to our predictions (we expected competition effects to exceed environmental filtering effects), our results indicate that no one ecological mechanism predominates over the other in determining community structure, at least in the Mediterranean region. The explanation here might be twofold. First, although behaviourally dominant ant species exclude other species from near their nests [52] and from high-value food resources that are spatially and temporally concentrated [34], the role of dominant ants influencing other species at the community level has recently been questioned [36–39]. In ant communities there are several compensatory mechanisms (e.g. thermal tolerance-behavioural dominance trade-off, dominance-discovery trade-off and its modulation by parasitoids) that may act on ant community organization by modifying the expected competitive outcome and thereby allowing coexistence [38,53]. In our study, all local communities come from the Mediterranean region, which is highly diverse in microhabitats with varying conditions, especially with respect to temperature [31]. In Mediterranean ant communities, dominant species usually have low thermal tolerance, whereas subordinate species have high thermal tolerance; therefore, subordinate species can forage at different times of the day or in different seasons compared with dominant species, leading to species coexistence and high diversity [31]. If so, we might only be able to detect what is no longer the result of past competition if we use a set of ecological traits that are hard to measure for a high number of species, such as physiological thermal tolerance, food resource discovery time and vulnerability to parasitoid attack. Whatever the case, our results support recent works that throw into doubt the role of competition as an important ecological mechanism structuring ant communities at the local scale [37,38]. We of course do not seek to generalize our conclusions at the local scale from the Mediterranean region to other regions. Indeed, assembly rules can clearly differ among biogeographic regions, as they probably provide contrasting evolutionary histories of ant faunas [54,55]. Thus, in much colder regions than the Mediterranean (i.e., boreal and alpine), where habitat heterogeneity is low and the abundance of behavioral ant dominant species is high, the biotic filter might play a more relevant role and thus competition might be the predominant force structuring local ant communities. [56–59]. Still, the role of competitive exclusion in the ant low-diversity systems typical from boreal and alpine forests remains unclear (39). And secondly, an alternative explanation might be that both environmental filtering and competition are similarly important at the local scale, and then their effect on community structure is compensated. This might only be possible if there is high spatial heterogeneity in microhabitats with contrasting environmental conditions. The fact that most of the few significant coefficients at the local scale were negative might suggest some role of environmental filtering in local Mediterranean communities. At any rate, conducting tests in local communities from other biogeographic regions might help to disentangle the relative role of environmental filtering and competition at small spatial scales.

Meanwhile, at intermediate spatial scales, i.e. at the biogeographic region scale, we found that environmental filtering is also the prominent mechanism structuring ant communities. In the Mediterranean, Continental, Atlantic and Boreal regions in particular, ant communities are predominantly structured by environmental filtering, with different effect magnitudes (i.e. different slopes of the relationship between species dissimilarity and species co-occurrence among biogeographic regions). Otherwise, in the Alpine biogeographic region, our results indicate a neutral model where no mechanism is more relevant than the other in the structuring of ant communities, or communities are randomly structured. This result suggests that differences in abiotic, biotic and historical conditions among biogeographic regions could be important in explaining differences in the dominant assembly rules in ant communities. On the one hand, the main biogeographic regions of Western Europe present varying climatic conditions [41] and are distributed across a clear latitudinal gradient (35 – 70°N). The relevance of environmental filtering might increase from low to high latitudes following a gradient of increased climatic variation (e.g. variation between the minimum and maximum temperatures) [60]. Accordingly, we found the role of environmental filtering increases following a clear latitudinal pattern (from the Mediterranean to the Boreal region).

On the other hand, and in contrast to the other regions, the Alpine region is distributed discontinuously across the study area, which might explain the highest beta functional community diversity in this region in relation to the other biogeographic regions of Europe [41]. The strong dispersal limitations of European ant species [61,62] might account for high functional turnover, probably due to divergent evolution. If account is taken of all these factors, the structure of Alpine ant communities might be determined by stochastic mechanisms, assuming that population dynamics do not depend on environmental characteristics and are primarily driven by ecological drift and dispersal [4].

With regard to our second aim to analyse whether the type of functional trait plays a relevant role in determining the relative importance of environmental filtering and competition, we corroborated our hypothesis. Our results indisputably indicate that trait type matters. However, we failed to predict the mechanism at work suggested by each trait type. This is because, as predicted, the analyses of the relationship between species pair co-occurrence and functional divergence with grouping of ‘ecological tolerance traits’, when significant, always revealed negative coefficients (environmental filtering effects). However, when the analyses were performed with grouping of ‘ecological niche traits’, significant relationships were associated with positive (competition effects) and negative (environmental filtering effects) coefficients. This does not support our hypothesis that ‘ecological niche traits’ are more related to competition processes. This is even clearer when looking at the patterns when analyses were performed with single traits, because the significant coefficients of single ecological tolerance traits were always negative; meanwhile, ecological niche traits displayed significant positive and negative coefficients. Whatever the case, classifying traits in ‘ecological tolerance traits’ seems to be appropriate in order to detect environmental filtering effects. In contrast, the balance for each single ecological niche trait can be either positive or negative, and this classification appears to be somewhat problematic. On the other hand, our results also demonstrate that, in general, performing assembly rules analyses with multi-trait grouping is good enough, since responses are the balance of different and contrasted responses. However, when conducted with a set of traits that clearly refer to only one ecological process, multi-trait analyses might be much more informative than pooling together traits of such diverse origin. Thus, the separate analyses of ‘ecological tolerance traits’ and ‘ecological niche traits’ provide more significant patterns than when pooling all of them together, given that opposite patterns can cancel each other out.

The analyses performed separating groups of traits fostered a more in-depth understanding of the assembly rules at work among the different biogeographic regions. We found no effect from any group of traits in the Alpine region. This might confirm that the lack of a significant relationship between species pair co-occurrence and functional dissimilarity is not due to compensation between the two mechanisms, but to randomly structured communities, probably as a result of historical factors and dispersal limitations, as previously suggested. Moreover, we found significant relationships for the Continental, Atlantic and Boreal regions for ‘ecological tolerance traits’ alone, and for the Mediterranean region for both ‘ecological tolerance’ and ‘ecological niche’ traits. These results suggest that the Continental, Atlantic and Boreal regions are mainly structured by environmental filtering, while competition can also be similarly relevant in the Mediterranean region. Ant activity and productivity are strongly related to temperature [29,35], and the effects of competition typically increase with increasing productivity [35,63]. Mean temperatures in the Continental, Atlantic and Boreal regions are much lower than in the Mediterranean region [Fig 2 in 41].

In short, environmental filtering is important for structuring European ant communities at large spatial scales, particularly at the continental scale and in most biogeographic regions. Competition could also play a role at intermediate spatial scales in those regions where environmental conditions are more favourable for ant productivity (i.e. the Mediterranean region). Meanwhile, stochasticity might be especially relevant in spatially discontinuous regions (i.e. the Alpine region). Different abiotic and biotic factors might therefore play an important role in determining which mechanism is more relevant in the structuring of ant communities at intermediate spatial scales. However, we failed to demonstrate the prevalence of any mechanism at the local scale in the Mediterranean region. We also conclude that the type of trait is important when seeking different assembly rules, and multi-trait grouping works well for traits that directly relate to environmental responses (i.e. ecological tolerance traits that respond to environmental filtering), but not for traits that are related to the way species exploit resources (i.e. ecological niche traits that respond to competition). Moreover, ecological niche traits might not be associated with competition, but also with other ecological processes such as equalizing fitness and facilitation [22]. We need more studies that address what directly links morphological and life-history traits with ecological processes, especially in animals, in order that we may disentangle the ecological processes that structure diversity at different spatial scales. Clearly, spatial scale of analysis, environmental context and chosen traits merit special attention in trait-based analyses of community assembly mechanisms.

## Supporting information

S1 Table Pearson correlation coefficients *r* among all traits. Significant coefficients are shown in bold (p<0.05). Abbreviations correspond to the following traits: Nqueen, number of queens; lnCS, colony size; BrCy, brood cycle; pSeed (proportion of seeds in diet), pInsects (proportion of insects in diet), pLiquid (proportion of liquid foods in diet); Diurn, diurnality; Ws, worker size.
(DOCX)

S2 Table Estimated coefficients from the analyses between co-occurrence and functional dissimilarity between species pair based on single traits at the local scale. Abbreviations: n, number of species pairs in each locality; n0, number of species pairs with co-occurrence = 0 in each locality; Nqueen, number of queens; lnCS, colony size; BrCy, brood cycle; Diet, diet; Diurn, diurnality; Ws, worker size; NA, not available coefficient. Note that binary co-occurrence analyses were only performed when at least six pairs of species depicted values of co-occurrence = 0. Significant coefficients (p<0.05), are shown in bold.
(DOCX)

S1 File Bibliographic sources from Table 1 to categorize traits as response or ecological niche traits. (DOCX)
