# Supplementary material for: The role of environmental vs. biotic filtering in the structure of European ant communities: A matter of trait type and spatial scale
Source: PLoS One. 2020 Feb 19;15(2):e0228625. doi: 10.1371/journal.pone.0228625 (PMC7029880; doi:10.1371/journal.pone.0228625)
Supplement: S1 Table — Significant coefficients are shown in bold (p<0.05). Abbreviations correspond to the following traits: Nqueen, number of queens; lnCS, colony size; BrCy, brood cycle; pSeed (proportion of seeds in diet), pInsects (proportion of insects in diet), pLiquid (proportion of liquid foods in diet); Diurn, diurnality; Ws, worker size. (DOCX) [file pone.0228625.s001.docx]

|  | Nqueen | lnCS | BrCy | pSeed | pInsects | pLiquid | Diurn |
| --- | --- | --- | --- | --- | --- | --- | --- |
| lnCS | **0.310** |  |  |  |  |  |  |
| BrCy | **-0.139** | **-0.168** |  |  |  |  |  |
| pSeed | **-0.188** | 0.036 | **0.231** |  |  |  |  |
| pInsects | 0.112 | **-0.189** | **-0.450** | **-0.405** |  |  |  |
| pLiquid | 0.070 | **0.139** | **0.198** | **-0.552** | **-0.539** |  |  |
| Diurn | **-0.230** | **-0.065** | **-0.458** | **-0.005** | **0.161** | **-0.142** |  |
| Ws | 0.003 | **0.335** | **-0.317** | **0.170** | -0.117 | -0.049 | **0.304** |
