# Supplementary material for: The role of environmental vs. biotic filtering in the structure of European ant communities: A matter of trait type and spatial scale
Source: PLoS One. 2020 Feb 19;15(2):e0228625. doi: 10.1371/journal.pone.0228625 (PMC7029880; doi:10.1371/journal.pone.0228625)
Supplement: S2 Table — Abbreviations: n, number of species pairs in each locality; n0, number of species pairs with co-occurrence = 0 in each locality; Nqueen, number of queens; lnCS, colony size; BrCy, brood cycle; Diet, diet; Diurn, diurnality; Ws, worker size; NA, not available coefficient. Note that binary co-occurrence analyses were only performed when at least six pairs of species depicted values of co-occurrence = 0. Significant coefficients (p<0.05), are shown in bold. (DOCX) [file pone.0228625.s002.docx]

|  |  |  | **Binary co-occurrence analysis** | | | | | | | **Co-occurrence strength analysis** | | | | | |
| --- | --- | --- | --- | --- | --- | --- | --- | --- | --- | --- | --- | --- | --- | --- | --- |
| **Locality** | **n** | **n0** | **Nqueen** | **lnCS** | **BrCy** | **Diet** | **Diurn** | **Ws** | **Nqueen** | | **lnCS** | **BrCy** | **Diet** | **Diurn** | **Ws** |
| loc.01 | 45 | 11 | 0.6291 | -4.5823 | 0.1542 | 0.7445 | -0.0645 | 2.0917 | 0.4604 | | -1.5548 | 0.4674 | 0.3929 | -0.0825 | 2.1075 |
| loc.02 | 120 | 40 | **1.1526** | **-5.2759** | 0.0703 | 0.2728 | 0.2022 | **-2.3185** | 0.1709 | | -0.0946 | 0.1332 | 0.4510 | 0.1447 | 0.1064 |
| loc.03 | 10 | 0 | - | - | - | - | - | - | -1.2039 | | 2.2763 | NA | -1.9384 | NA | **-9.2679** |
| loc.04 | 66 | 29 | **3.8874** | -2.6141 | -0.9395 | 2.0346 | **-1.3416** | **-3.2931** | **-0.9656** | | 1.5737 | -0.0811 | -0.6045 | -0.3302 | **-1.5363** |
| loc.05 | 6 | 0 | - | - | - | - | - | - | -0.7517 | | 2.7007 | NA | -1.6098 | NA | 8.5905 |
| loc.06 | 21 | 12 | 2.7726 | -6.2594 | -0.5596 | 4.5746 | NA | 7.7535 | 1.0767 | | -5.6266 | -0.6960 | -1.2369 | NA | -1.5616 |
| loc.07 | 21 | 0 | - | - | - | - | - | - | -0.0841 | | 2.1958 | -0.6307 | 1.2660 | -0.2909 | -0.9021 |
| loc.08 | 21 | 6 | -0.7951 | -4.9216 | NA | 2.3043 | -1.3863 | -4.5769 | -0.0848 | | -1.4478 | NA | -0.6292 | 0.6860 | -3.2183 |
| loc.09 | 120 | 40 | 0.0992 | -0.0654 | 0.3610 | 1.3722 | 0.1728 | 1.4018 | 0.1192 | | 0.8348 | 0.0055 | -0.5901 | 0.1627 | 0.0382 |
| loc.10 | 28 | 7 | -0.6404 | -10.132 | NA | 3.4739 | < 0.0001 | 1.6498 | 0.2795 | | -.4263 | NA | -0.7721 | 0.4557 | 0.2599 |
| loc.11 | 66 | 20 | **2.2943** | -4.8572 | 1.6635 | -1.8917 | -0.6678 | -0.6074 | -0.1620 | | **-3.6630** | 0.2830 | 0.3884 | 0.1127 | -0.1451 |
| loc.12 | 55 | 3 | **-** | - | - | - | - | - | 0.0159 | | 1.0777 | 0.3709 | 0.5200 | -0.4191 | 0.3951 |
| loc.13 | 21 | 3 | - | - | - | - | - | - | 0.3774 | | 1.7086 | NA | **-1.3137** | NA | **-1.2834** |
| loc.14 | 28 | 4 | - | - | - | - | **-** | - | 0.4014 | | 0.4431 | NA | -0.4730 | -0.0782 | **-1.0763** |
| loc.15 | 21 | 6 | 0.8267 | 10.492 | 0.9163 | 0.2898 | 0.9163 | -0.6026 | 0.4040 | | 1.0752 | -0.0384 | -1.1362 | -0.0384 | -1.2078 |
| loc.16 | 21 | 0 | - | - | - | - | - | - | **-1.1898** | | 1.3156 | 0.5563 | -0.4524 | 0.2458 | -1.7270 |
| loc.17 | 28 | 1 | - | - | - | - | - | - | 0.2565 | | 0.7692 | 0.4772 | 0.8046 | 0.1183 | -2.0016 |
| loc.18 | 36 | 2 | - | - | - | - | - | - | 0.5846 | | 0.9184 | 0.2584 | -0.6406 | 0.2312 | -0.2906 |
| loc.19 | 15 | 6 | -1.8330 | -1.5853 | -1.2528 | < 0.0001 | -0.9163 | -4.982 | -0.4595 | | 1.5735 | 0.5200 | -0.6382 | -0.2771 | -0.2498 |
| loc.20 | 55 | 35 | 0.9067 | -3.2073 | 0.1611 | 0.1355 | -0.3725 | 0.3064 | -0.1749 | | -1.4090 | **0.6064** | **-1.2584** | 0.3893 | 0.4620 |
| loc.21 | 36 | 20 | -0.5242 | -2.6601 | 2.6931 | 0.4352 | -0.4520 | -0.6929 | -0.0411 | | -0.8413 | -0.3906 | 0.2434 | -0.5100 | -0.6572 |
| loc.22 | 28 | 4 | - | - | - | - | - | - | -0.4452 | | -0.6430 | 0.2977 | -0.0113 | 0.1289 | -0.3155 |
| loc.23 | 66 | 3 | - | - | - | - | - | - | 0.0725 | | -0.5449 | **0.5303** | -0.2888 | 0.1749 | -0.7027 |
| loc.24 | 45 | 13 | 0.8265 | -4.1010 | -0.8755 | 2.8445 | -0.4055 | 1.9540 | 0.1400 | | -0.3063 | -0.1454 | 0.1772 | 0.0770 | 0.8219 |
