# Supplementary material for: The role of environmental vs. biotic filtering in the structure of European ant communities: A matter of trait type and spatial scale
Source: PLoS One. 2020 Feb 19;15(2):e0228625. doi: 10.1371/journal.pone.0228625 (PMC7029880; doi:10.1371/journal.pone.0228625)
Supplement: S1 File — (DOCX) [file pone.0228625.s003.docx]

**Supporting Information S2.**

**(A) Ecological tolerance**

Arnan, X. et al. 2014. Ant functional responses along environmental gradients. - J. Anim. Ecol. 83: 1398–1408.

Hölldobler, B. and Wilson, E. O. 1990. The ants. - Harvard University Press.

Parr, C. L. et al. 2017. GlobalAnts : a new database on the geography of ant traits (Hymenoptera: Formicidae). - Insect Conserv. Divers. 10: 5–20.

Number of queens

Hölldobler, B. and Wilson, E. O. 1977. The number of queens: An important trait in ant evolution. - Naturwissenschaften 64: 8–15.

Colony size

Beckers, R. et al. 1989. Size, communication and ant foraging strategy. - A J. Entomol. 96: 239–256.

Linksvayer, T. A. and Janssen, M. A. 2008. Traits underlying the capacity os ant colonies to adapt to disturbance and stress regimes. - Syst. Res. Behav. Sci. 26: 315–329.

McGlynn, T. P. and Kirksey, E. S. 2000. The effects of food presentation and microhabitat upon resource monopoly in a ground-foraging ant (Hymenoptera : Formicidae) community. - Rev. Biol. Trop. 48: 629–642.

Retana, J. et al. 2015. A multidimensional functional trait analysis of resource exploitation in European ants. - Ecology 96: 2781–2793.

Brood cycle

Kipyatkov, V. E. 1993. Annual cycles of development in ants: diversity, evolution, regulation. - Proc. Colloq. Soc. insects in press.

**(B) Ecological niche**

Retana, J. et al. 2015. A multidimensional functional trait analysis of resource exploitation in European ants. - Ecology 96: 2781–2793.

Diet

Cerda, X. I. M. et al. 1997. Thermal of transitive hierarchies in disruption ant communities Mediterranean. - J. Anim. Ecol. 66: 363–374.

Cerdá, X. et al. 1998. Prey size reverses the outcome of interference interactions of scavenger ants. - Oikos 82: 99–110.

Retana, J. and Cerdá, X. 1994. Worker size polymorphism conditioning size matching in two sympatric seed-harvesting ants. - Oikos: 261–266.

Diurnality

Cerda, X. I. M. et al. 1997. Thermal of transitive hierarchies in disruption ant communities Mediterranean. - J. Anim. Ecol. 66: 363–374.

Cros, S. et al. 1997. Spatial and temporal variations in the activity patterns of Mediterranean ant communities. - Ecoscience 4: 269–278.

Savolainen, R. and Vepsäläinen, K. 1989. Niche Differentiation of Ant Species within Territories of the Wood Ant Formica polyctena. - Oikos 56: 3–16.

Worker size

Gibb, H. and Parr, C. L. 2010. How does habitat complexity affect ant foraging success? A test using functional measures on three continents. - Oecologia 164: 1061–1073.

Retana, J. and Cerdá, X. 1994. Worker size polymorphism conditioning size matching in two sympatric seed-harvesting ants. - Oikos: 261–266.

Retana, J. et al. 2015. A multidimensional functional trait analysis of resource exploitation in European ants. - Ecology 96: 2781–2793.
